# Commentary on Joseph et al.’s (2022) “Transition Experiences of Indian Nurses Into Australian Mental Health System”

**DOI:** 10.1177/10436596221128778

**Published:** 2022-10-08

**Authors:** Mohammed Al-Sheikh Hassan

**Affiliations:** 1 Faculty of Health and Life Sciences, De Montfort University, Leicester, UK; 2 University Hospitals of Leicester NHS Trust, UK

The study of Joseph et al. (2022) explored the transition experience of Indian-trained nurses into Australian mental health care settings. The study employed hermeneutic phenomenology as the approach, guided by van Manen’s (1990) framework. The study included a purposive sample of 16 participants who were recruited via advertising and snowballing techniques. Data were collected via in-depth individual interviews and analyzed thematically. Initially, I want to emphasize that this commentary article does not aim to comment on or critique the methodology and methods of Joseph et al. (2022) but to comment on the study’s aim, findings, and discussion.

To begin with, the authors did not state whether the nurses were originally practising mental health nursing in India before moving to Australia. I find it crucial to report the work history of the nurses to understand the nurses’ transition journeys properly. The authors only stated that the nurses were initially trained in India without highlighting if they had previous clinical experience in India, particularly in mental health. In contrast, other studies clarified the issue of home country nursing experience in exploring and understanding the experience of transitioning to and practising nursing in other foreign countries (Kishi et al., 2014).

The study’s outcome included four themes: *living in dual culture*, *loneliness*, *discrimination*, and *feeling incomplete*. Although the study aimed to explore the transition experience of Indian-trained nurses into Australian mental health nursing, only one theme, discrimination, indirectly touched on the issue of mental health nursing. Consequently, the title and aim of this study could be misleading due to the generic focus of its findings and discussion. Specifically, all other study themes highlighted general life or personal experiences outside the clinical setting. In addition, the only theme that illustrated aspects of mental health nursing was about how the nurses were “*discriminated*” by being physically and verbally assaulted by patients with mental health conditions or being discriminated against by their native colleagues. Still, the authors did not provide a clear linkage between being a mental health nurse and the discrimination of other colleagues in the care setting. Moreover, the included direct quotations of participants supporting the discrimination theme indicate different scenarios, which are heavily about workplace violence.

In opening the study’s discussion section, the authors provided a different aim, stating, “This study sought to understand the transition experiences of overseas-trained nurses from India and their current situation while living in Australia.” (p. 45). This aim completely conflicts with the original aim and the study’s title, ignoring the issue of mental health nursing. In addition, most of the discussion section is about socio-cultural aspects of the experience, while minimal elements of mental health nursing practice were discussed. Specifically, the discussion section lacks a clear demonstration of the study’s outcome. For example, the authors state: “findings from this current study suggest additional stressors faced by migrant Indian nurses working within mental health area” (p. 46). However, the narrative is not supported by relevant literature about the issue so that readers can position this specific outcome. This issue is evident in the article, where the authors avoided the core aim of the research, transitioning to mental health, and focused the work on presenting and discussing generic transition experiences.

Again, the authors state that the literature includes zero studies about the experience of overseas nurses working in mental health. However, they did not focus their study on presenting findings about mental health nursing transition experiences. Therefore, the work of Joseph et al. (2022) can be seen as similar to and consistent with other studies that presented general transition experiences of foreign-trained nurses anywhere in the world (Dicicco-Bloom, 2004). Finally, the authors concluded that the nurses struggled to understand the language and workplace practice model, which was not presented in the study’s findings. In conclusion, I agree with the authors that the literature lacks examples of foreign-trained nurses’ transition experiences from a speciality-specific perspective. Therefore, nurse researchers are encouraged to focus on studying foreign-trained nurses’ experiences of transitioning to specific specialities such as critical, emergency, and trauma care as many generic transition experience studies are widely available at Australian (Chun Tie et al., 2018) and global levels (Moyce et al., 2016).
